# Toward Improved Treatment and Empowerment of Individuals With Parkinson Disease: Design and Evaluation of an Internet of Things System

**DOI:** 10.2196/31485

**Published:** 2022-06-09

**Authors:** Liran Karni, Ilir Jusufi, Dag Nyholm, Gunnar Oskar Klein, Mevludin Memedi

**Affiliations:** 1 Centre for Empirical Research on Information Systems Örebro University School of Business Örebro Sweden; 2 Department of Computer Science and Media Technology Linnaeus University Växjö Sweden; 3 Department of Medical Sciences, Neurology Uppsala University Uppsala Sweden

**Keywords:** Internet of Things, wearable technology, Parkinson disease, patient empowerment, objective measures, self-assessment, self-management, web interface

## Abstract

**Background:**

Parkinson disease (PD) is a chronic degenerative disorder that causes progressive neurological deterioration with profound effects on the affected individual’s quality of life. Therefore, there is an urgent need to improve patient empowerment and clinical decision support in PD care. Home-based disease monitoring is an emerging information technology with the potential to transform the care of patients with chronic illnesses. Its acceptance and role in PD care need to be elucidated both among patients and caregivers.

**Objective:**

Our main objective was to develop a novel home-based monitoring system (named EMPARK) with patient and clinician interface to improve patient empowerment and clinical care in PD.

**Methods:**

We used elements of design science research and user-centered design for requirement elicitation and subsequent information and communications technology (ICT) development. Functionalities of the interfaces were the subject of user-centric multistep evaluation complemented by semantic analysis of the recorded end-user reactions. The ICT structure of EMPARK was evaluated using the ICT for patient empowerment model.

**Results:**

Software and hardware system architecture for the collection and calculation of relevant parameters of disease management via home monitoring were established. Here, we describe the patient interface and the functional characteristics and evaluation of a novel clinician interface. In accordance with our previous findings with regard to the patient interface, our current results indicate an overall high utility and user acceptance of the clinician interface. Special characteristics of EMPARK in key areas of interest emerged from end-user evaluations, with clear potential for future system development and deployment in daily clinical practice. Evaluation through the principles of ICT for patient empowerment model, along with prior findings from patient interface evaluation, suggests that EMPARK has the potential to empower patients with PD.

**Conclusions:**

The EMPARK system is a novel home monitoring system for providing patients with PD and the care team with feedback on longitudinal disease activities. User-centric development and evaluation of the system indicated high user acceptance and usability. The EMPARK infrastructure would empower patients and could be used for future applications in daily care and research.

## Introduction

Parkinson disease (PD) is a chronic degenerative disorder that causes progressive neurological deterioration with profound effects on the affected individual’s quality of life [1-4]. It affects 1 out of 100 people aged >60 years, and its prevalence has doubled since 2005 [5]. The progression of the movement disorder is often accompanied by the development of neuropsychiatric deficits in the later stages. The characteristic fluctuations of various neurological symptoms become increasingly pronounced over the years [6]. Clinicians and patients find it challenging to establish a treatment strategy based on the patient’s ability to plan and self-administer levodopa to temporarily counteract the motor deficits. Over time, it often becomes difficult to stabilize the fluctuations in the neurological symptoms, and patients may need more advanced treatments based on specialist assessments [7]. Current disease management strategies rely on scheduled in-hospital assessments and periodic contacts with expert physicians. However, in Europe, 44% of newly diagnosed people with PD do not meet neurologists with an interest and expertise in PD despite recommendations to do so [1]. Patients also have limited access to specialist nurses and occupational therapists owing to resource limitations or patient’s disabilities [5,8,9]. In addition, periodical snapshot assessments performed during in-hospital visits carry the possibility of over- or underestimation of the longitudinal disease burden and cannot reliably describe the situation these patients face in their home environment [4,5,9-11].

Therefore, there is a substantial interest in developing home-based care models for PD [5,12]. The main goal of this approach is to provide autonomy and self-efficacy for self-management through an increased understanding and interpretation of the associations among disease symptoms, daily life activities, and treatment [13,14]. Technological advancements can largely contribute to improvements in the home-based care of people with PD [4,12,15-18]. For instance, several device-aided treatments are available for managing motor fluctuations in people with PD, such as deep brain stimulation, infusions of apomorphine or levodopa [19,20] and microtablets of levodopa [21]. Such advanced treatments may be tailored to the needs of each individual. The Task Force on Technology of the International Parkinson and Movement Disorder Society has called for tighter collaboration among clinicians, researchers, patients, and engineers to achieve this goal by developing home-based integrated measurement and closed-loop therapeutic systems with high patient adherence [12]. The task force also envisioned that this strategy could lead to the adoption of clinical-pathophysiological phenotyping and early detection of milestones in disease progression, improve subgroup targeting of patients for future testing of disease-modifying treatments, and identify objective biomarkers to improve longitudinal tracking of impairments in clinical care and research [12].

The possibility of home-based continuous monitoring of PD symptoms is now possible using new technologies available today, such as the Internet of Things (IoT), in the form of wearable sensors to capture fluctuating disease activities [16,22], potentially serious incidents such as falls or freezing of gait [23-29], or assistance with the medication strategy [30]. There is also an expectation that the technology-based objective measurements may, in the future, replace or amend the current practice of disease severity assessment and follow-up that mostly rely on patient-reported outcomes and clinical scales [14,30,31]. For instance, in our previous publications, we demonstrated good validity, reliability, and responsiveness to treatment changes using objective measures based on multiple wearable sensors for motor function assessment [32,33].

Despite these technological advances, it is still unclear how these tools could be translated into a better patient self-management and mutual care plan or how they could improve the overall health outcomes [34]. Some important questions that remain to be answered include what clinical or patient needs should be addressed, by which technology, and how these systems could be deployed in patients’ homes to maximize treatment benefits and compliance, while minimizing the risk of disruption to daily life [11,12,14]. Based on these considerations, the Movement Disorder Society Task Force on Technology has recently deliberated that future research and development should be based on the identification of clinically meaningful targets for modification instead of a pure technology-driven selection of measurement end points [12].

Targeting patients by providing feedback about their disease activities and their related self-management is a promising strategy for PD. Prior publications almost exclusively focused on patient home assessment and measurements as tools for clinical decision-making and targeted clinicians as end users. Home monitoring of disease activities can increase the extent and quality of self-engagement, which in turn can potentially increase patient satisfaction and lead to positive changes in the health-related quality of life and outcomes [12,13,31,35]. This symptom-driven self-management strategy can be especially successful for people with PD who are knowledgeable about the disease functions [36]. It is presumable that such patient-targeted feedback efforts would empower people with PD with noticeable differences in the efficacy of self-management, patients’ communication with the care team, and improved health-related outcomes [36].

We recently published the outlines of an IoT-based system (named EMPARK) aimed at empowering people with PD and their care team [32,33]. The system is based on home monitoring, with the aim of collecting longitudinal data for patient self-assessment and clinical decision-making. We identified patient empowerment as an important primary end point of information and communications technology (ICT) interventions in people with PD. In a subsequent publication, we described the design of a patient interface prototype for EMPARK along with its initial assessment with people with PD [32,33]. The interface was designed to visualize symptom and medication information collected by an IoT-based system comprising a mobile technology (tablet), electronic dosing device, wrist sensor, and bed sensor. The results indicated that patients found visual feedback on motor function, sleep, medication compliance, meal intake timing, and their relationship to self-medication as a useful tool that could improve their daily self-management and mutual care planning with their physicians.

Improving patient empowerment has been a key concept behind the development of EMPARK. A retrospective evaluation of its ICT design against the ICT for patient empowerment model (ICT4PEM) [37], together with the results of the patient interface evaluation, indicated that EMPARK has a strong potential to empower its primary users, the patients [32,33].

Although the EMPARK system has not yet been deployed in practice, in this study we demonstrate its system architecture, implementation, and evaluation of user interfaces. As EMPARK is a project under development, our demonstration builds on our prior results regarding the data and interface architecture of the system [32,33]. As part of this demonstration, we also provide evidence of how the EMPARK system could empower patients and members of the care team.

## Methods

### Overall Study Design and Theory

The EMPARK system was developed based on the principles of design science research [38], user-centered design [39,40], and the overall target of patient empowerment. As an initial step, a set of long-term goals was agreed upon by the authors and through iterative consultation with clinical experts in PD. These goals also served as the foundation for the development of the overall and specific requirements for system design. These requirements were regarded as the core tasks of the EMPARK system development.

After a thorough study of the literature and several iterations with experts, we developed the ICT4PEM, a novel framework to support future ICT interventions that primarily aim to improve patient empowerment [37]. To the best of our knowledge, the ICT4PEM is the only framework that provides clear, International Organization for Standardization–style definitions for core empowerment characteristics (control, psychological coping, self-efficacy, understanding, legitimacy, and support) and allocates them with project-specific, well-defined targets for ICT interventions. This framework defines the core characteristics of the empowerment concept as the primary target of ICT interventions. It also defines a set of empowerment consequences as potential targets of indirect intervention, such as expressed patient perceptions, behavior, clinical outcomes, and health system effects. ICT4PEM stratifies interventional design into strategies, narrowed further into ICT services, and finally, specific ICT tools. To ensure the goals of empowerment, targeting ICT4PEM requires that each ICT service within a project must be allocated to specific empowerment domains with strict definitions. Finally, ICT4PEM is intended to be used for project evaluations with the aim of demonstrating the effects on patient empowerment or its consequences [37].

We applied the ICT4PEM to evaluate the ICT design of the EMPARK system in terms of its relevance and strength for patient empowerment. This was possible by identifying well-defined patient empowerment characteristics within the ICT4PEM that could be easily identified as the primary targets of the EMPARK system interventions. Consequently, we categorized the ICT interventions of EMPARK by strategies, as described in ICT4PEM, and allocated these strategies and ICT interventions to the relevant empowerment characteristics. This analysis of the EMPARK design with the ICT4PEM framework was based on our results from an extensive evaluation of the patient mock-up interface [32,33] by patient users. In addition, evaluation of the clinician interface revealed additional feedback on the patient-empowering potential of EMPARK from clinician users.

### Study Variables

#### Selection of Study Variables

PD is a complex and multimodal neurological disorder that affects the patients’ quality of life. Therefore, the system collects multidimensional data to represent the overall health status of the patients [12,40]. The concept of overall health status, based on consultations with participating expert neurologists and patients, was defined as a data set that incorporates both objective and self-reported measures of ongoing disease activities and, regarding the disease-specific symptoms, includes both motor and nonmotor functional parameters.

Furthermore, decisions regarding the inclusion of variables were made by examining the set of requirements shown in Textbox 1.

The final selection of individual variables and relevant technologies for home implementation and data acquisition was based on prior literature and recommendations from the Movement Disorders Society Task Force [12] and by consultation with expert physicians and patients. Subsequently, this selection was refined based on the evaluation of mock-up interfaces for patients and physicians.

**Requirements.**

- Potentially empower the patients if they receive feedback on it.
- Improve understanding of their disease and its relationship with their daily activities and self-management decisions.
- Improve self-efficacy and control of daily disease management.
- Improve the overall feeling of support in their self-management and communication with the care providers.
- People with Parkinson disease, except those with very advanced disease, are capable of self-reporting (regarding self-reported data only).
- Availability of reliable technology for its objective measurement, and that the technology is nonintrusive in the home environment and nondisrupting regarding the patient’s regular daily activities.
- Capable of stable wireless data transfer.
- Preferentially sensor technology with the capacity to provide data for multiple end points (core data).
- The least possible maintenance dependent.
- Data are assessed as clinically relevant by expert physicians.
- Data are assessed as scientifically relevant for future studies regarding their clinical validity.

#### Calculation of Study Variables

##### Overview

The EMPARK system collects health information from the home environment of patients. The system summarizes the objective data (medication compliance, sleep and motor function) as well as subjective data (meal intake time, physical exercise and quality of life) and calculates the corresponding scores per calendar day. All the scores were produced in the range of 0 (low) to 100 (high). Most of these parameters have been previously described elsewhere [32]. Nevertheless, we provide a list of these parameters with their definitions and a brief description of the method of calculation.

##### Medication Compliance Score

Using the planned and delivered medication intake times, the medication compliance score was calculated by first calculating the deviation (in minutes) as depicted in the following equation:



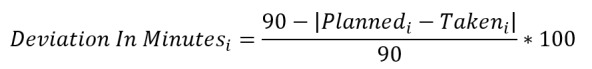



where *Planned_i_ i*s the time of a planned medication intake and *Taken_i_* is the time of a delivered medication intake. This resulted in scores ranging from 0 to 100. The final medication compliance score per day was calculated using the following equation:



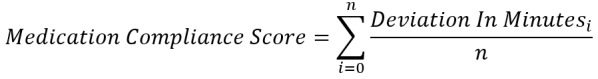



where *n* is the total number of medication intake occasions per day. Patients can take an extra dose to either replace an intended dose that was initially missed or to take another dose. Extra doses administered within 30 minutes before or after a planned time were considered as replacement and omitted in calculations, whereas, for other extra doses, the compliance score was set to the highest score, that is, 100.

##### Sleep Score

This was obtained by using the time information recorded by the bed sensor. A sleep score was calculated based on 3 individual components: sleep duration, habitual sleep efficiency, and sleep disturbance. The components were derived based on the Pittsburgh Sleep Quality Index and the work of Williams and Cook [41].

To calculate sleep duration, the system first calculated the differences (in total hours) between the time when the bed was occupied and when it was empty and calculated the cumulative hours being in bed:



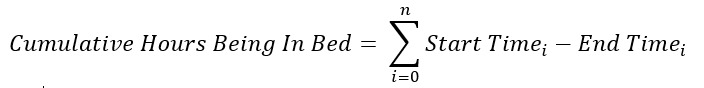



where *n* is the total number of recorded intervals when the individual was in bed. The following rules were applied to calculate sleep duration:

- *IF Cumulative Hours Being In Bed* > 7 *THEN Sleep Duration* = 0
- *IF Cumulative Hours Being In Bed* > 6 *AND Cumulative Hours Being In Bed* < 7 *THEN Sleep Duration* = 1
- *IF Cumulative Hours Being In Bed* > 5 *AND Cumulative Hours Being In Bed* < 6 *THEN Sleep Duration* = 2
- *IF Cumulative Hours Being In Bed* < 5 *THEN Sleep Duration* = 3

The second component was the habitual sleep efficiency score, which was calculated by first calculating the ratio between total hours being in bed with interruptions and total hours being in bed without interruptions using the following equation:



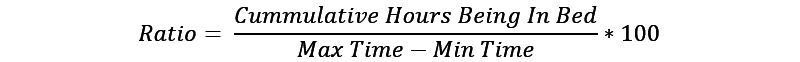



where *Min Time* is the time when the patient went to bed for the first time during a particular day and *Max Time* is the time when the patient left the bed the next day. The habitual sleep efficiency score was calculated according to the following rules:

- *IF Ratio* ≥ 85 *THEN Habitual Sleep Efficiency* = 0
- *IF Ratio* > 75 *AND Ratio* <85 *THEN Habitual Sleep Efficiency* = 1
- *IF Ratio* > 65 *AND Ratio* <*75 THEN Habitual Sleep Efficiency* = 2
- *IF Ratio* < 65 *THEN Habitual Sleep Efficiency* = 3

The third component that was calculated was sleep disturbances score using the following steps:

Step 1: Count the number of interruptions during sleep, which resulted in *Total Number Of Interruptions* score.

Step 2: Apply the following rules:

- *IF Total Number Of Interruptions* = 0 *THEN Sleep Disturbances* = 0
- *IF Total Number Of Interruptions* = 1 *THEN Sleep Disturbances* = 1
- *IF Total Number Of Interruptions* = 2 *THEN Sleep Disturbances* = 2
- *IF Total Number Of Interruptions* ≥ 3 *THEN Sleep Disturbances* = 3

Step 3: Calculate the final sleep score using the following equation:



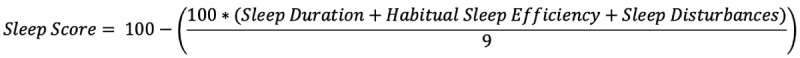



##### Motor Function Score

The wrist sensor-based system produced scores every 2 minutes for bradykinesia and dyskinesia. To summarize bradykinesia and dyskinesia scores per day, initially the mean values were calculated.

##### Meal Timing Compliance Score

An algorithm calculated the alterations from the recommended meal intake time (30 minutes before and 60 minutes after levodopa medication intake) to calculate the score:



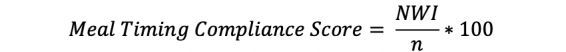



where *NWI* is the number of reported occasions when meal intake time was within the recommended interval of 30 minutes before and 60 minutes after medication intake, and *n* is the total number of reported occasions of meal intake during a day.

##### Physical Activity Score

The score per day was calculated using the following equation:



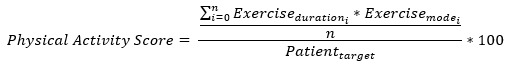



where *Exercise_duration_* is in minutes, *Exercise_mode_* is the reported physical exercise mode (1: boxing, dancing, running, and swimming; 2: bicycling and gym; and 3: walking), *Patient_target_* is the individual patient target ranging from 0 to 100, and *n* is the total number of physical exercise occasions reported per day. As there may be more than one exercise occasion, the algorithm first calculates an average score per day followed by its normalization using the patient target. For instance, if a patient reported 2 exercise occasions during a particular day with a target of 80 with the following data: for the first occasion the patient reported exercise mode of 1 and duration of 10 minutes, for the second occasion the patient reported exercise mode of 2 and duration of 25 minutes then the physical exercise score for the day would be 75 × {[10 + (2×25)]/80×100}.

##### Quality of Life Scores

This was derived from each day the patients use a custom application to answer 9 health-related questions (Table 1) taken from the 8-item Parkinson Disease Questionnaire [42] and the EQ-5D-3L (European Quality of Life 5 Dimensions 3 Level) health questionnaire [43]. Inclusion or exclusion of the questions from the 8-item Parkinson Disease Questionnaire and EQ-5D-3L was performed in collaboration with clinicians at Uppsala and Örebro University Hospitals. The first 8 questions are scored on 3 levels: no problems (level 1), some problems (level 2), and extreme problems (level 3). The last question is about the overall health when the patients are asked to rate their health on a visual analog scale ranging from 0 to 100 where 0 is the *worst imaginable health* and 100 is the *best imaginable health*.

**Table 1:** Quality of life items and source questionnaires.

Item number	Item	Questionnaire
1	Mobility	EQ-5D-3L^a^
2	Personal care	EQ-5D-3L
3	Daily activities	EQ-5D-3L
4	Pain or discomfort	EQ-5D-3L
5	Worry or depression	EQ-5D-3L
6	Concentration difficulties	PDQ-8^b^
7	Communication difficulties	PDQ-8
8	Painful cramps or spasms in the muscles	PDQ-8
9	Overall health	EQ-5D-3L

^a^EQ-5D-3L: European Quality of Life 5 Dimensions 3 Level.

^b^PDQ-8: 8-item Parkinson Disease Questionnaire.

### Elicitation of the User-Specific Requirements

Requirements related to patient and clinician interfaces were gathered through discussions with the end users based on the principles of user-centered design [39].

The development of these requirements for the patient interface has been discussed elsewhere [32,33]. This section provides an outline. The patient interface had 4-stage patient- and expert-based development cycles that revealed 8 categories of information to be presented to the patients.

Regarding the clinician interface, a broad set of requirements was identified during 5 group discussions or brainstorming design [44] sessions between the authors, medical specialists in neurology with strong clinical backgrounds and substantial experience with the management of people with PD, and nurses from the neurology departments at the university hospitals of Uppsala and Örebro, Sweden. During the first session, medical doctors and nurses were informed about the overall system architecture of the proposed EMPARK system. During this session, the participants outlined the requirements that they anticipated the clinician interface would offer them. These requirements were documented by taking notes and later elaborated upon by the design team. The design principles of the clinician interface were derived from these sessions.

### Evaluation of the Users’ Interfaces

The mock-up patient interface was subject to extensive user-centric evaluation as previously published [32]. We provide a summary of the evaluation process. We created simulated patient scenarios to be used during the development and evaluation of the patient interface. Each patient case was based on a database developed by clinical domain experts. The database was in an Excel file format, which was subsequently loaded into the server, and presented to the patients as test subjects during the late development and evaluation phases of the patient interface as published earlier. These mock-up scenarios were later used to determine user acceptance [33]. After heuristic evaluation of the initial patient interface, subsequent evaluation was performed by requesting patients to perform tasks on the mock-up interface that mimicked daily life tasks based on realistic patient data. The patients needed to use the capacities of the interface to summarize disease activities by week or day, alternatively showing data at a higher temporal resolution.

Similarly, simulated clinical scenarios of PD with corresponding data sets were developed by collaborating expert physicians to evaluate the clinician interface. The evaluation was performed with the help of 2 professionals (a neurologist and nurse) from the Uppsala University Hospital. After a brief introduction to the functionalities of the interface, the users had to complete different tasks using the prototype clinical interface representing the simulated clinical scenario, following a semistructured interview. The participant were instructed to comment loudly on their thoughts during the completion of the tasks according to the *think aloud* methodology [45]. The task-based evaluation session was documented by the investigators during a one-to-one session with the evaluator and was later used for further refinement of the prototype clinician interface. This first task-based evaluation step intended to assess the overall usability of the interactive functions and visual solutions of the interface and included simple instructions. In the subsequent step, the participants were asked to complete 3 complex tasks related to specific clinical case scenarios to test the functionalities of the prototype and the clinicians’ understanding. This was video recorded using a screen-capture software for later documentation and analysis (Multimedia Appendix 1).

We also conducted semistructured interviews (Multimedia Appendix 2) with the same 2 participants during the same session. The development of questions and evaluation of the responses that were part of the semistructured interviews followed the methodology heuristic evaluation and questions [46] and the Computer Usability Questionnaire [47]. The questions were developed through iterative brainstorming meetings among the researchers. The responses were audio recorded for subsequent transcription and analysis.

### Ethics Approval

Ethics approval from the ethics review authority will be applied for at the clinical trial stage according to Swedish law. However, this paper communicates the early evaluation of the clinical interface that was using dummy data with no personal information. This interpretation of the lack of requirement for ethics approval for this step has been checked with the internal policy of our universities.

## Results

### Requirement Elicitations

In accordance with the overall study design, the first stage of EMPARK development was the determination of its major goals. The list and definitions of these goals are as follows:

- System development: to develop an IoT system comprising sensors and mobile technology to deliver home monitoring of objective motor function, medication use, and patient-reported symptoms and outcomes.
- Patient empowerment: to empower patients and improve their self-management through a better understanding of their disease with the help of feedback from the system.
- Clinician support: to provide physicians with relevant and useful information derived from the system regarding symptoms, treatment response, and individual patient coping strategies for better clinical assessment and treatment strategy.
- Improvement and enablement of research: to establish the EMPARK system as a tool for researchers to better understand the complexity of the disease and to develop and monitor new drugs.

The core tasks of the EMPARK system development were defined as described in the *Methods* section to accomplish the major goals. These included selection of patient-related data for longitudinal acquisition, development of visual interfaces to facilitate follow-up of the health status of people with PD by clinicians, as well as to provide feedback to patients on their data.

### User Interface Requirements

The mock-up patient interface was functional at the time of the development of the clinician interface. The requirements elicited for the patient interface were also found to be satisfactory by the participating medical professionals. These requirements have been published previously [32]. Briefly, these included motor function, sleep, medication compliance, meal intake times, physical exercise, and a subjective assessment of the day as parameters. Patient requirements for data representation included routines of an ideal day, effect of meal timing on medication, single day parameter overview, and revealing the effects of motor functions on various other parameters and daily routines. Detailed and aggregated data were table-based, with the possibility of narrowing down the selection.

The clinician interface aims to provide access to aggregated and analyzed data of clinical interest. Furthermore, the patient interface can be shared with the physician or care team for shared decision-making and elaboration of a mutual care plan. The main objectives behind the development of a clinician interface were the elaboration of a web-based application for the analysis and representation of EMPARK data. These objectives were defined based on the following list of specific requirements elicited by iterative consultations with the care team:

- Provide the care team with data on longitudinal fluctuations of disease symptoms, daily patient activities, and self-medication.
- Help clinicians discover disturbances, limitations of disease self-management, or aberrations from the care plan.
- Facilitate communication and co–decision-making with patients about treatment based on objective and longitudinal data on disease activities and self-management.
- Help the care team foster patient empowerment based on objective data.

Physicians and nurses also requested access to and visualization of raw data in addition to integrated summaries. A further requirement was an overview graph representing the daily summary with scores from different variables, such as medication, mealtime, exercise, self-assessment, sleep score, and the 2 motor function scores, such as those for bradykinesia and dyskinesia. Other requests were a flexible and interchangeable time axis for the score results with temporal zoom function, allowing discoveries of temporal associations between different disease activities and treatment and the possibility of selecting variables for representation.

### EMPARK System Architecture

#### Overview

**Figure 1:**
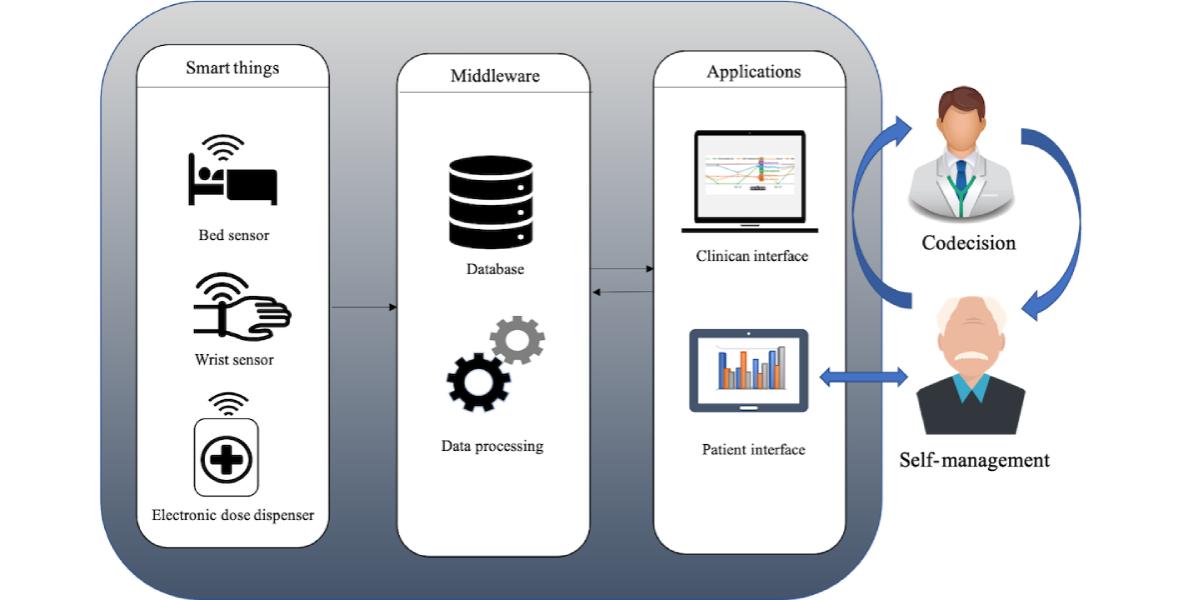
EMPARK architecture.

The EMPARK system (Figure 1), which is currently under development, leverages IoT technologies to empower people with PD and assist clinicians in making informed decisions. The system is deployed at the patient’s home and consists of a set of commercial IoT devices: a wrist sensor that provides measures of bradykinesia and dyskinesias; a medication dosing device that dispenses individualized doses of levodopa based on schedules defined by clinicians; a bed sensor attached to the bed frame that captures data about sleep patterns (time and quality); and a tablet app to collect self-reported data on physical activity, health-related questions, and meal intake time. Data were collected continuously 24×7 and fed into a database. The data processing layer aggregates and analyzes the provided data. A detailed description of the architecture is provided in our previous study [32]. The system has 2 interfaces: a tablet-based app for the patient and a web-based interface for the clinician. Although the data foundation for both interfaces is identical, the use and presentation of the data are tailored to the user’s needs.

The functional EMPARK system has 2 intercalated application cycles: one for patient empowerment and self-management and one for codecision and facilitation of mutual care plan development (Figure 1). The self-management cycle represents home-based disease monitoring and direct feedback of information to the patient through the patient interface. Self-management becomes cyclic when the feedback provided modifies patient self-management, which consequently alters the results of home monitoring. The codecision cycle represents cyclic changes in patient self-management based on the patient’s interaction with the care team. As the source of the clinical decision-making is home monitoring, the latter is positioned in the middle of the 2 circles.

#### Hardware and Software Specifications

The data processing layer, which consists of algorithms for processing data from different sensors and a custom software for summarizing the scores, is written in the C# programming language. The web application (clinician interface) was developed in Python 3.6 using Dash Plotly for visualization. The tablet app (patient interface) was written in C# using Microsoft Xamarin for Android, and SQLite was used to access the data. The principles of the Model-View-View Model Prism architectural design pattern were followed for the development of the patient interface app to ensure high-level cohesion.

#### User-Specific Functionalities

#### Patient Interface Functionalities

This section is based on our 2 previous publications on the EMPARK patient interface. The first publication describes user-centered design and evaluation processes and provides a detailed description of interface functionalities [32]. The second publication elaborates the factors that influence user acceptance [33]. As part of the ongoing development of EMPARK, and based on the results of the evaluation, some changes have been implemented in the patient interface. These changes were intended to improve the overall functionality of the interface and user acceptance. Briefly, the previously published version of the patient interface allowed users to select from 3 major representation modes. The start page showed color-coded icons representing the mean values for the last 14 days of the 6 main parameters: overall day score, medicine score, motor function score, physical exercise, meal, and sleep scores. Good, average, and bad scores were defined as >70, 30 to 70, or <30 in value and color coded as green, gray, or orange, respectively. Patients could navigate by clicking on a parameter leading to a 3-week summary of the selected score, showing the daily, color-coded results. From here, patients could select a specific week to see each day of the week as a horizontal graph with graphical representation of the sleeping hours, times of meals and exercises, and the overall daily score in color coding. Patients could also select a day view with a similar timeline-based visualization of the scores (with turn on and off for the presentation of each variable). This page allowed the user to identify temporal associations among elements of self-management (ie, medicine intake or mealtime) with outcome parameters, such as motor function score or sleep quality.

**Figure 2:**
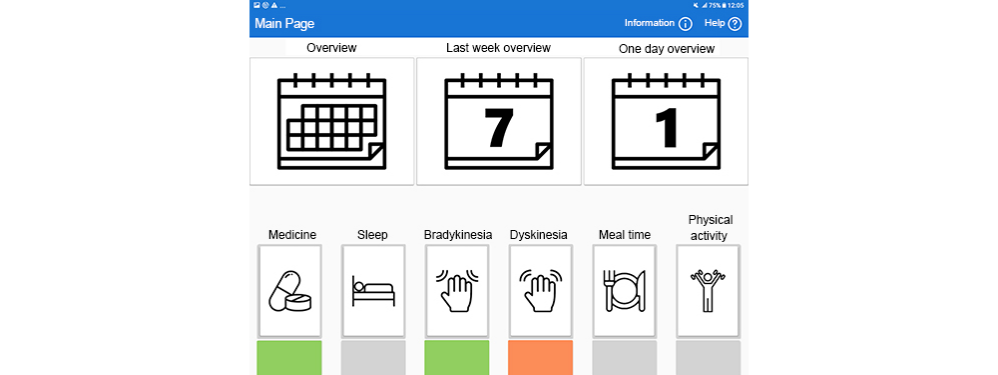
Navigation (home page; translated from Swedish to English).

**Figure 3:**
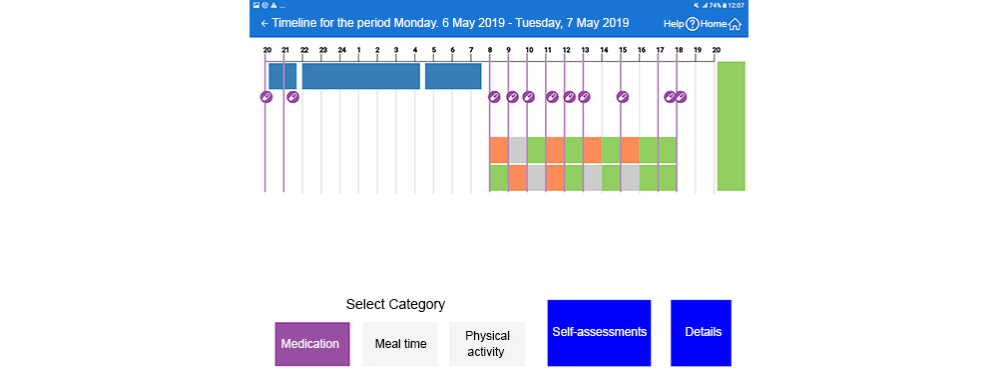
One-day view with separated bradykinesia and dyskinesia motor function scores (translated from Swedish to English).

**Figure 4:**
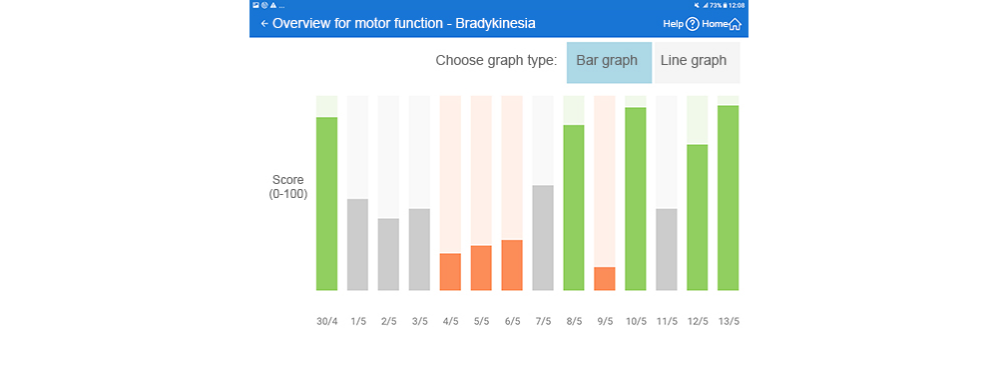
Bar or line graph (translated from Swedish to English).

**Figure 5:**
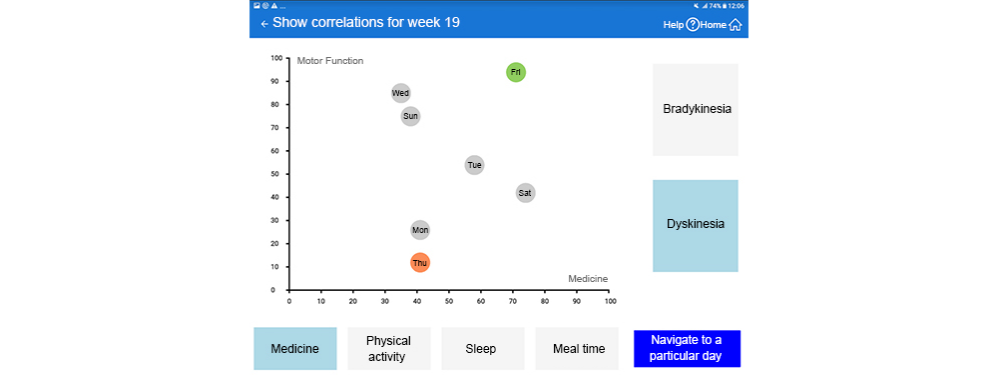
New correlation view of motor function score versus medication compliance score (translated from Swedish to English).

In the revised version of the patient interface, the following changes have been made:

1. The navigation from the home screen was improved by new functional and design elements that allowed the user to select and open a 2-week view, a 1-week view, and a 1-day view, as well as open views for the 6 scores according to their actual preference (Figure 2).
2. The 1-day and 1-week views and the motor function (bradykinesia and dyskinesia) scores are presented separately, in contrast to the prior presentation of their averaged values (Figure 3).
3. Similarly, the temporal associations among the 6 different scores (medication compliance, sleep, bradykinesia, dyskinesia, meal compliance, and physical activity over the last 2-week period) became easier to realize, as we completed this view with additional bar and line graphs (Figure 4).
4. Finally, to facilitate the discovery of correlation among the relevant parameters, the correlation view was modified. It is now possible to change the y-axis to the 2 motor function scores (Figure 5).

#### Clinician Interface Functionalities

The development of the clinician interface application was based on specific user requirements as previously described.

**Figure 6:**
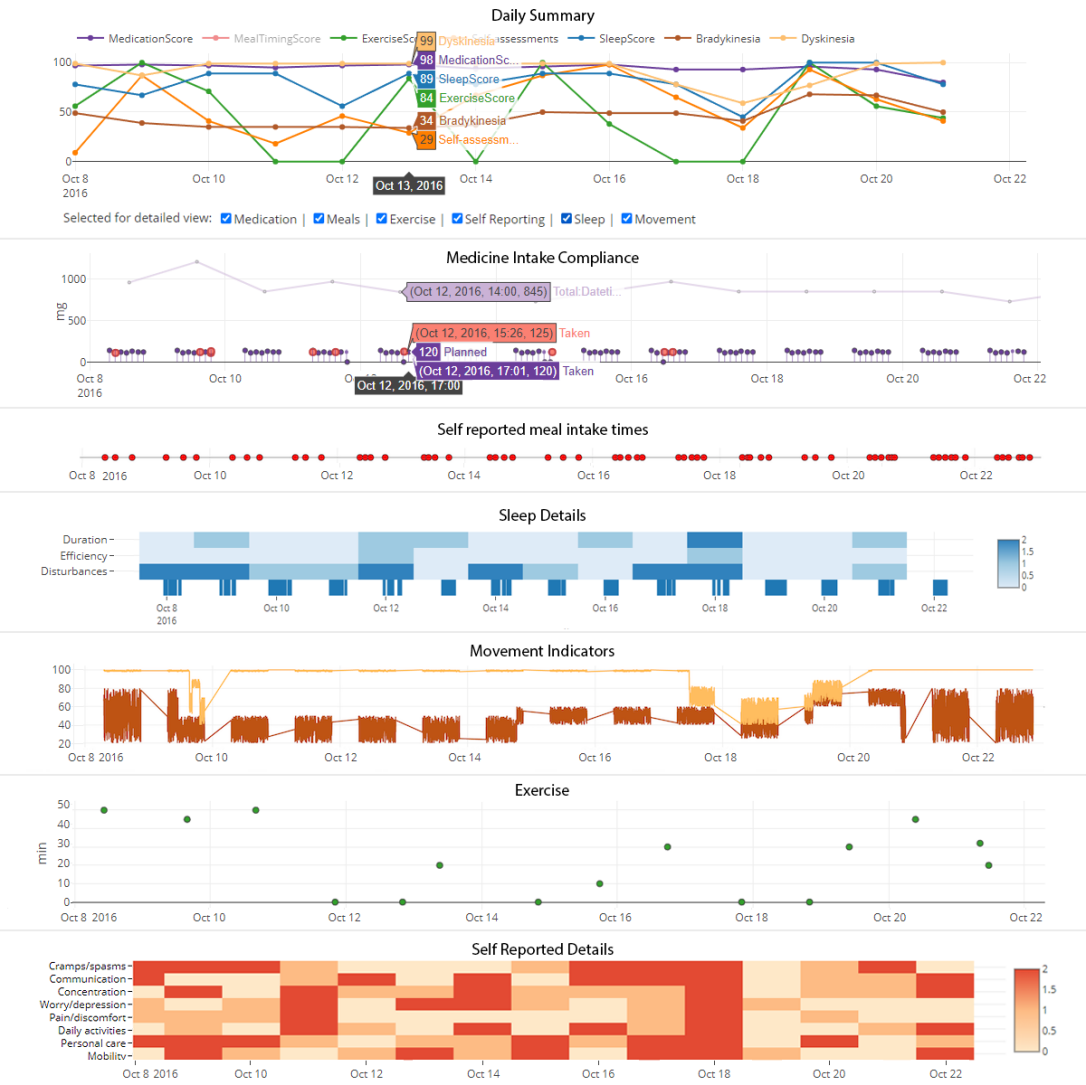
A snapshot of the clinician interface. The first graph shows a summary of the scores per day during the last 2 weeks. In the detailed views, users can select or deselect variables: medicine intake compliance, self-reported meal intake times, sleep details, movement indicators, exercise, and self-reported details as a complement to the first graph.

To enable rapid patient status assessment, information in the clinician interface is displayed and ordered using a top-down approach with a general overview of the patient’s scores and assessments. The main page displays the patient’s Daily Summary graph (Figure 6). The Daily Summary is a collection of graphs of the selected variables that can be updated interchangeably: medication score, mealtime score, exercise score, self-assessment, sleep score, and the 2 motor function scores (bradykinesia and dyskinesia). The horizontal axis displays the date, and the vertical axis displays the patient’s score on a scale ranging from 0 to 100, where 0 is considered as the worst score and 100 is regarded as the best. The user can add or remove variables by selecting or deselecting them.

The raw data and more detailed information for each component are shown in speared graphs, allowing the user to subsequently switch between components by using the checkbox list: (1) medicine intake compliance, (2) self-reported meal intake times, (3) exercise, (4) self-reported details, (5) sleep details, and (6) movement indicators.

Some of the main components are the composites of several variables. Self-reported details are composed of several subjective variables (cramps or spasms, communication, worry or depression, pain or discomfort, daily activity, personal care, and mobility) that follow a simple scoring system (0-2), where 0 represents the worst result and 2 represents the best possible result. For the most comprehensive overview of all these parameters along the time scale, we chose a color-coded tile for representation. Sleep details represent sleep duration, efficiency, and disturbances in separate graphs. Movement indicators show bradykinesia and dyskinesia as variables. Following the requirements of the end users, these graphs represent the time plot of the raw wrist sensor data, which neurologists are already familiar with.

We used standard time-series data visualization techniques [48]. Some facets were changed slightly to accommodate specific tasks. The most prominent is the medicine intake compliance chart. The grayed-out line plot shows a cumulative dosage taken per day. The gray circles with horizontal lines represent the planned medicine intake, whereas its vertical positioning corresponds to the planned dosage value. The purple circles indicate the actual dosage taken for the planned intervals. Patients sometimes receive extra doses as indicated by the red circles.

Clicking on any designated point over the graph prompts a pop-up window that displays the exact values for that point of time. Moreover, it is possible to zoom in and out by selecting a section of interest. This action will affect all graphs simultaneously.

### Results From User Interface Evaluation

The patient interface, as previously published, was subject to extensive evaluation [32,33]. Overall, the patients found the visualizations clear and easy to understand and could successfully perform the tasks.

Here, we demonstrate the results of clinician interface evaluation.

The first task series (Multimedia Appendix 1) included instructions to assess the direct usability of the interface functionalities. The users were introduced to the interface shortly before completing these tasks but without specific descriptions and demonstrations of all user functionalities in a visualization of the clinical parameters. Hence, the completion of the tasks was largely based on the intuitive application of the interface. Both users accomplished these tasks with only minor delays and without the need for an intervention from the evaluator. The users also assessed the functionalities as easy to comprehend and in accordance with the expected level of information technology literacy and experiences of an average clinician or nurse user.

The second task series (Multimedia Appendix 2) aimed to evaluate the functionality of the interface in solving complex clinical questions using task-based evaluation. Specifically, the tasks were developed to be a logical continuation of a problem-solving process in which the users were subject to discovering points of interest in the clinical case scenarios.

Notably, there was a marked difference in the response times between the physician and nurse evaluators. The former completed each task faster by using the functionalities of the interface in a more intuitive way. Similarly, we observed an easier comprehension of the complexity of the represented clinical data by physicians compared with the nurses. The physician completed these tasks with only minimal loud comments that did not reveal any experienced difficulties.

In contrast, the nurses required a substantially longer time to solve the same tasks. The delay in task completion by the nurse evaluator was also accompanied and well explained by the loud comments about the difficulties. In agreement with our own observations, these comments suggested that the nurses experienced more difficulties in comprehending the complex clinical data.

The observations from the analysis of the task completion performance were further elucidated through subsequent semistructured interviews (Multimedia Appendix 3). As the answers for one specific question often included information that corresponded to the other questions, we recategorized the responses by analyzing each sentence separately. To increase the validity of the responses, we analyzed the transcripts for eventual contradictions; for instance, contradictory responses from the same interview participant at different time points in the interview. However, we were unable to identify these statements.

The overall combined assessment of the interview transcripts and observations made by task completion led to the identification of key areas of user interest. These areas were also identified as the main pathways for future development of the system.

We summarized our findings by demonstrating relevant sentences from the evaluators for each defined area (Table 2). The responses from the interview participants were categorized by topic and content analyses. Incongruence among the interview participants is not shown separately but is instead highlighted by the coexistence of both satisfactory and critical comments regarding the same area. Specifically, our nurse participant was overall more critical of the interface dashboard, and these critical comments were mostly about the design of the interface.

**Table 2:** Summary of user evaluation results for the clinician interface.

	Benefits	Suggested development or modification
Intuitiveness	Enables clinical staff by providing otherwise-unavailable clinical data of great potential relevance; empowers physician-patient relationship; enables potential research in data analysis at large.	Automated correlation analysis among different parameters of the system.
Design	Simple overview and organization of information. Easy to accomplish most tasks at once (physician’s view).	Improvement of overall design; time-scale harmonization among parameters and zooming function; making data more clearly understandable.
Empowering the health care provider and usefulness in clinical practice	Novel and useful information content; objective clinical decision-making; comparison with patient’s subjective experiences; can improve codecision making; allows early distant identification of patient with nonadherence or unsatisfactory disease control; identification of area for intervention in management; research possibilities by analyzing EMPARK data at a population level.	Motor function (wrist sensors) data presentation to be harmonized with available commercial systems (eg, Parkinson KinetiGraph) [49]; relevant treatments beside levodopa; further patient-derived information to explain fluctuations of parameters; same visual platform (ie, patient interface) for codecision making.
Empowering the patient	Can improve codecision making.	Patient messaging (also to empower the provider).

Both users appreciated the novelty and intuitiveness of the EMPARK system regarding its potential for making important disease-relevant data readily available to the clinical practice. Similarly, both users highlighted that in their routine clinical practice, they lack information that the EMPARK system provides. The users found the clinician interface simple enough to accomplish simple tasks that simulated the daily applications. The physician user had no criticism and rapidly completed the tasks without assistance. The nurse found some potential flaws in the current design of the interface that could cause potential problems with later widespread application. He found that the data presentation was clear enough. Both users commented on the lack of time-scale harmonization among the displayed parameters, which made the discovery of associations more difficult. One of the users encouraged the development of automated correlation analysis to support the discoveries of potential causative associations. The clinician suggested that the format of motor function data visualization should be in agreement with what they are accustomed to when analyzing the motor data collected by similar systems. The physician would also welcome the incorporation of additional patient-derived data to the EMPARK, such as commenting possibilities for patients to explain their special pattern of symptoms or report on life events (eg, watching an exciting movie that may have caused dyskinesias or delays in medication intake), which could have an impact on either the symptoms or self-management. However, it was also added by both users that such extensions may backfire at the level of patient adherence to EMPARK, as simplicity is often the key for long-term patient cooperation.

### Patient Empowerment and the EMPARK system

Using the ICT4PEM [37], we identified the patient empowerment characteristics (PEMs) defined by the model that have clear relevance to and are in accordance with the main goals of EMPARK system development. These PEM characteristics, along with their definitions from ICT4PEM, are listed as follows:

- Control: The ability by which an individual can decide about their level of engagement in the health care process and participate in decisions regarding alternative treatment options, including when these are performed by professionals.
- Understanding: the potential use of the information a patient has regarding their own *health status*, the *diseases*, and the function of the actual and possibly available *health care processes*. Notably, understanding, in our definition, represents the patient’s capacity to apply *knowledge* in the specific and individual context of the disease and health care provision. *Information* and its availability to the patient serve as a basis for understanding. Consequently, neither knowledge nor information is sufficient as a characteristic of *empowerment*, although both should be considered as prerequisites for understanding.
- Self-efficacy: the sum of cognitive and physical capabilities possessed by the patient that can be used for self-care.

We were also able to identify several ICT strategies as described by ICT4PEM, which bear obvious relevance to the ICT interventions of EMPARK. In accordance with ICT4PEM, the ICT interventions must target one or several PEM characteristics. Subsequently, we allocated the selected ICT interventions of EMPARK to these intervention categories as described below:

- Feedback
  Feedback about symptoms, medication, sleep, and activities on a daily and weekly basis
- Monitoring
  IoT-based home monitoring of disease symptoms, sleep, and self-medication
  Self-reporting of physical activities and mealtimes
  Self-reporting of subjective health-related variables
- Communication
  Providing a visual platform, with disease data used by both clinicians and patients, facilitating mutual communication for co–decision-making.
- Analysis
  Scoring of symptoms
  Scoring of physical activities
  Analysis of self-medication
  Scoring of sleep
  Correlation analyses

The sessions with patient organization and questionnaires used to evaluate the design confirmed that these ICT strategies would be welcome for the improvement of the 3 empowerment characteristics we want to improve. However, the degree of effect on empowerment requires evaluation studies that specifically target these characteristics after real-life deployment of EMPARK in clinical studies.

## Discussion

### Principal Findings

This study presents the latest results from the ongoing system development and evaluation of a novel IoT-based home monitoring system (EMPARK) for people with PD. The EMPARK system represents a unique conceptual approach for both its design and long-term goals. To our knowledge, EMPARK is a unique ICT intervention among people with PD, as it aims to provide patients with feedback about their disease symptoms and disease management. Accordingly, patient empowerment through various means is one of the main goals of the overall research project. The EMPARK system also aims to support the care team through the dynamic visual representation of previously unavailable clinical information and, as a long-term goal, contributes to the academic research.

Primarily, EMPARK is an ICT system that relies largely on IoT tools deployed in the patient’s home environment as a source for input data, complemented by patient-related outcome parameters of disease activities and self-management. The objectively measured parameters include continuous daily measures of time spent in bed, motor functions, and medication (levodopa). The self-reported parameters were the time of meals, physical activities, and health-related quality of life questionnaires. As we published information previously, EMPARK synthetizes these parameters with various level of complexity to achieve *daily scores* of sleep quality and duration, motor function, medication compliance, meal timing compliance, and physical activities. Daily scoring of these parameters enables an easy-to-comprehend visualization for patients, along with the potential to provide physicians or nurses with detailed data for deeper analysis. We must emphasize that we have not yet achieved the final goal of implementing a functional and full-scale EMPARK ICT infrastructure in daily practice. However, our current and previous findings provide evidence that the main components of the system, such as databases, computing, and visual interfaces, are almost fully developed and evaluated. Our results from the evaluation of the interfaces highlight directions for potential future developments. For instance, the users suggested automated correlation analysis for discoveries of potential causative associations among the different parameters and to use them either as part of the patient feedback or as clinician support.

Multiple lines of evidence suggest that the EMPARK system will be empowering for people with PD. The ICT design of the EMPARK system followed a user-centered design and patient-centric development. This included iterative steps of development and evaluation with respect to both the patient and clinician interfaces. We recently published a novel framework (ICT4PEM) for ICT interventions, with patient empowerment as the main target [37]. Importantly, the lessons learned through patient-centric design and evaluation of EMPARK made an important contribution to our recognition of the urgent need for such a framework. Retrospective analysis of the ICT design through the principles of ICT4PEM clearly indicates that the system will empower patients. The conceptual elements of patient empowerment can be readily identified as the system targets. Similarly, ICT interventions within the system fit to those ICT intervention strategies described by the ICT4PEM. Reviewing our previous findings from patient interface evaluations indicates a high level of patient acceptance of the technology and its intuitive use for solving real-life problems common in disease self-management. The content and visual design of the patient interface were well appreciated by the evaluators and in agreement with the previously set requirements during the early developmental stage. Notably, patient feedback from the evaluation led to improvements in the functionalities. We could explain over 80% of the observed interuser variabilities in intention to use by the usual determinants, such as sociodemographic and technology-associated factors. Patient interviews were in accordance with these findings, implicating a high potential for improvement in patient satisfaction, disease control, understanding, and self-efficacy. The latter 3 conceptual elements are also important elements of the patient empowerment paradigm [37]. Taken together, the results of the multistep evaluation with the mock-up patient interface provide us with strong confidence to believe that the selected variables for feedback are neither too little nor too much for improving self-management and ensuring targeted improvements in patient empowerment.

The system also sets the support for the care team as a main project goal. In this study, we describe the user-centric development of a clinician interface and the results of its user-centric evaluation using multiple methodologies. The professional management of patients with PD relies largely on periodic *snapshot* outpatient visits. This renders the care team members to be reliant on patients’ memories of their past symptoms in an attempt to obtain a longitudinal picture of the ongoing disease activities and their self-management. Our findings clearly show that care team members, both the physician and nurse, often regard such information as unreliable because of recall bias and find it insufficient for decision-making. Frequent and high-amplitude fluctuations in PD symptoms further emphasize the need for reliable longitudinal follow-up [7,12]. We believe that the application of IoT tools, such as wrist and bed sensors in the patient’s home, will be a nonintrusive way to introduce novel data with clinical relevance in the decision-making process [50]. Importantly, the physicians and nurses who participated in the development or the evaluation of the clinician interface share our thoughts. Evaluating care team members think that the parallel availability of information about self-management and disease symptoms will lead to discoveries about the causative associations among them. In addition, our evaluators regarded it crucial for clinical decision-making, as such discoveries could be the basis of a new way of communication and agreement about care plans between the care team members and the patients. In accordance with the suggestions from our clinical interface evaluators, we plan to use the patient interface as the primary tool for shared decision-making for patients and care team members. We obtained evidence regarding the usability and appropriate visual design of the clinician interface. At the same time, we also observed differences that most probably relate to the differential tasks and background knowledge of the different care team members. Clinician interface, in its current form, seems to favor physicians compared with nurses with respect to comprehension and intuitive use. Nevertheless, the low number of evaluators should be regarded as a limitation as such differences in user experience may also be explained by other factors. We are currently undertaking efforts to expand the number of participants in the clinician interface evaluation to better understand the determining factors of user acceptance.

Research support is also one of the long-term goals of the EMPARK system. We now present clear support for such potential as part of the evaluation of the clinician interface. The evaluators of the clinician interface support our view that the EMPARK infrastructure, with a special focus on the newly defined scores of daily PD-related activities, could serve the purposes of observational or randomized controlled trials in the future. Our findings strongly support the incorporation of the scored parameters developed by the system into clinical trials, which can be justified by their relevance to the clinical decisions, lack of availability via other sources, and the patients’ own judgment about their usefulness. Such trials could help to establish the role of a parameter scoring system in the daily care of patients with PD, either after or partially in parallel with the previously described stages of implementation. In addition, it would also allow the scientific evaluation of a novel care model for patients with PD, with a focus shifted from snapshot controls to longitudinal assessment and mutual care plan implementation.

### Limitation and Future Work

There are several limitations with respect to the generalizability of our results, which are not only with regard to the potential user acceptance of a clinician interface in a large-scale trial.

We are aware that the implementation of the EMPARK system is crucial for assessing long-term user acceptance. Evaluators of the clinician interface and professionals with extensive experience in PD care consider the progressive neurological deficits of patients with PD as a potential risk for a diminishing system implication in the home environment over a longer period. Although we agree, we believe this risk is low considering our results that indicate a high level of acceptance and assessed usefulness among patient evaluators. Similarly, a conclusive assessment of patient empowerment and enhanced self-management, which are our main goals, awaits real-life implementation of the EMPARK system.

To address these issues and evaluate strategic goals, we are currently undertaking steps toward the experimental implementation of the EMPARK system in a real-life milieu. Early, limited implementation (ie, single-institute evaluation) can follow the design research principles with the potential of an ongoing, on-demand, smaller adjustment of the system architecture. However, the main assessment of long-term goals, such as the effect of the EMPARK system on patient empowerment, overall health status, or auxiliary effects on health care use, would require overall system stability during a larger clinical trial. We suggest that such a clinical trial should also address the role of the scored parameters as tools for daily clinical practice in PD care. Implementation at a larger scale would result in the accumulation of a large amount of clinical data. We believe that the great potential of such a database for auxiliary research can facilitate the implementation of the EMPARK system by health care providers.

### Conclusions

The EMPARK system is a novel IoT-based home monitoring system for providing patients with PD and team members with feedback about disease activities. EMPARK is unique in its primary aim of providing patients with feedback on disease symptoms and self-management, consequently enhancing patient empowerment. We describe the user-centric development and evaluation of the system’s clinical interface and show results indicating its high usability in clinical management and shared decision-making with patients. We also provide extensive evidence of how the EMPARK system infrastructure would empower patients and suggest future application in PD-related research. Our results represent the last phase in development before the implementation of EMPARK in a real-life environment.

## Appendix

Multimedia Appendix 1 First task-based evaluation.

Multimedia Appendix 2 Second task-based evaluation.

Multimedia Appendix 3 Semistructured interview questions.

## Abbreviations

EQ-5D-3L: European Quality of Life 5 Dimensions 3 Level
ICT: information communication technology
ICT4PEM: ICT for patient empowerment model
IoT: Internet of Things
PD: Parkinson disease
PEM: patient empowerment model
