# Supplementary material for: Toward Improved Treatment and Empowerment of Individuals With Parkinson Disease: Design and Evaluation of an Internet of Things System
Source: JMIR Form Res. 2022 Jun 9;6(6):e31485. doi: 10.2196/31485 (PMC9227793; doi:10.2196/31485)
Supplement: Multimedia Appendix 1 [file formative_v6i6e31485_app1.docx]

### Appendix 1: 1st Task-based evaluation

1. At the Daily Summary graph, add to view “meal timing score”
2. At the Daily Summary graph, remove from view the “meal timing score”
3. Enlarge a graph.
4. Minimize a graph by double clicking
5. Open to view the graphs: Meals, Exercise, Self-reporting, and Sleep.
6. Do you understand the color coding of the Self-reporting and Sleep graphs?
