# Supplementary material for: Toward Improved Treatment and Empowerment of Individuals With Parkinson Disease: Design and Evaluation of an Internet of Things System
Source: JMIR Form Res. 2022 Jun 9;6(6):e31485. doi: 10.2196/31485 (PMC9227793; doi:10.2196/31485)
Supplement: Multimedia Appendix 2 [file formative_v6i6e31485_app2.docx]

### Appendix 2: 2^nd^ Task-based evaluation

Task #1

1. Based on information presented in the Daily Summary graph, can you find out what caused the dyskinesia score to become worse on October 9 as compared to other days?
2. Look at the dyskinesia score for Oct 9 by zooming on the Movement Indicators view (Note: select Oct 9 and Oct 10). Why do you think the dyskinesia score is getting worse after 15:00, improving around 16:00, and then getting worse after 19:00?

Task #2

1. In the Daily Summary view, select Medication Compliance Score and deselect Bradykinesia and Dyskinesia”. Can you find out why the patient got a Medication Compliance Score of 80 on Oct 21?
2. Zoom out all the views. Select Meals in detailed views and deselect Movement. Add Meal Timing Score to the Daily Summary view. Can you see why the patient has a Meal Timing Score of 0 on Oct 15, 50 on Oct 13, and 100 on Oct 11?

Task #3

Zoom out all the views. Select the following detailed views: Exercise, Self-reporting, Sleep, and Movement. In the Daily Summary view, select Exercise Score, Self-assessments, Sleep Score, Bradykinesia, and Dyskinesia.

- Can you explain what happened with these scores during Oct 17 – Oct 19?
- Do you see a correlation between Exercise, Sleep, Self-Reporting, and Movement Indicators (Bradykinesia, Dyskinesia)?
- What may have caused improvements in scores on Oct 19?
- Do you see any trend in Sleep and Self-Reporting data during these three days?
