# Supplementary material for: Toward Improved Treatment and Empowerment of Individuals With Parkinson Disease: Design and Evaluation of an Internet of Things System
Source: JMIR Form Res. 2022 Jun 9;6(6):e31485. doi: 10.2196/31485 (PMC9227793; doi:10.2196/31485)
Supplement: Multimedia Appendix 3 [file formative_v6i6e31485_app3.docx]

### Appendix 3: Semi-structured interview questions

1. Do you think the clinician interface is intuitive?
2. What do you think about the general design of the clinician interface (such as text size, colors etc.) and the organization of the information on the screen?
3. What do you think about the ease of completing the tasks?
4. Do you think this system can improve your understanding of your Parkinson’s patient?
5. Do you think that this system can facilitate your communication with the patients and possibly improve their empowerment?
6. Do you think the system has all the functions and capabilities you expect it to have?
7. Overall, are you satisfied with the system? Any other comments?
